# Fermented Dairy Products, Musculoskeletal and Mental Health in Older Adults: is There Evidence to Support Benefits that go Beyond Those of Non-Fermented Dairy Products?

**DOI:** 10.1007/s00223-024-01291-4

**Published:** 2024-09-20

**Authors:** M. Papageorgiou, M. Lyrakou, A. Kyriacou, E. Biver, M. Yannakoulia

**Affiliations:** 1 Division of Bone Diseases, Geneva University Hospitals and Faculty of Medicine, University of Geneva, 1205 Geneva, Switzerland; 2 Department of Nutrition and Dietetics, Harokopio University, 17671 Athens, Greece

**Keywords:** Fermented dairy products, Osteoporosis, Fractures, Sarcopenia, Cognition, Mental health

## Abstract

Fermented dairy products have recently gained popularity due to their purported health benefits, nevertheless, their role in ageing remains uncertain. This narrative review aims to evaluate evidence from observational (prospective) and interventional studies on the potential benefits of fermented dairy product consumption for musculoskeletal and mental health in older adults. Additionally, it seeks to determine whether any observed benefits surpass those of non-fermented dairy products and to identify directions for future research. Prospective studies support either favourable or neutral associations of fermented dairy products with outcomes of musculoskeletal health or neutral associations with mental health outcomes, whilst it remains unclear if the benefits observed with fermented dairy products go beyond those of the non-fermented dairy foods. Few interventional studies suggest overall favourable effects of yogurt and cheese on musculoskeletal health in older adults but given their small number (N = 6) and heterogeneity, they do not allow a clear assessment or definitive recommendations for fermented dairy intake. Interventional studies reporting mental health outcomes are largely lacking for this age group (N = 1). Given the very limited evidence for the effectiveness of fermented dairy products, future well-designed prospective and randomized controlled trials are needed to better understand their benefits (especially compared to those of non-fermented dairy foods), their characteristics and the quantities required to offer protection against musculoskeletal and/or mental health ageing.

## Introduction

Nutrition is one of the main modifiable factors that may promote healthy aging and delay age-related diseases [1]. In this context, several dietary patterns, individual food groups and nutrients have been investigated for their potential benefits [2] and fermented foods could not be absent from this research field. Fermented foods are defined as foods and beverages made through controlled and desired microbial growth and enzymatic conversions of major and minor food components [3, 4]. Fermentation has been used since ancient times, mainly to preserve foods, but also to improve their organoleptic properties. Nowadays epidemiological evidence indicates that dietary patterns rich in these products can benefit human health, reduce disease risk, and enhance longevity and quality of life [5, 6].

Most well-designed observational and clinical studies on fermented foods have investigated the associations/effects of fermented dairy products (i.e., yogurt, cheese, kefir and other fermented milk products) on various health outcomes including gastrointestinal health, cardiometabolic health, and cancer risk [7, 8]. Nevertheless, there is limited evidence on their effects on conditions and diseases that are related to functional ability of older adults *i.e.,* musculoskeletal, and mental health outcomes. A systematic review showed some protective effects of dairy foods, including fermented dairy foods, against frailty/sarcopenia in community-dwelling older adults [9]. However, findings related to cognitive decline were inconsistent and contradictory [9]. However, this work included studies published by 2017 and it did not isolate the effects of fermented dairy products. In relation to bone health, there are several narrative and/or systematic reviews and meta-analyses on the effects of total and/or fermented dairy products [10–14], These were not limited to older individuals, and it remains uncertain if their conclusions also apply to this age group. The need for understanding the relation between fermented dairy products and musculoskeletal/mental health outcomes specifically in older adults is underpinned by: (i) their increased susceptibility to declines in musculoskeletal and mental health, (ii) their decreased capacity to absorb and metabolize nutrients effectively due to aging-related changes in the digestive system and overall metabolism, (iii) their increased requirements for several nutrients (e.g., proteins, calcium, vitamin D), (iv) their often-reduced energy intake and/or poor nutritional status (i.e., pre-existing nutrient deficiencies, making them more dependent on dietary sources for maintaining musculoskeletal/mental health) and (v) the presence of comorbidities, medication use and changes in lifestyle parameters including reductions in physical activity, which may exacerbate bone and muscle loss and/or cognitive deficits [15–18].

**Fig. 1:**
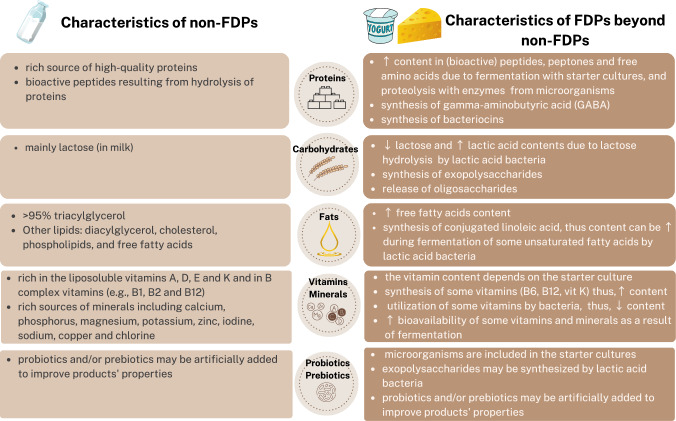
Characteristics of non-fermented and fermented dairy products. *FDPs* fermented dairy products, *GABA* gamma- aminobutyric acid, ↑ higher, ↓ lower

Although fermented dairy foods are being promoted due to their purported health benefits and are often preferred by consumers [19, 20], direct comparisons of the health effects of fermented vs. non-fermented dairy products are largely lacking. Based on epidemiological studies and plausible physiological mechanisms, several mechanisms have been proposed to support the hypothesis of more favourable health effects of fermented dairy products (vs. non-fermented) (Fig. 1). These include their enhanced nutrient profile as a result of the fermentation process (fermented dairy products are rich in proteins, vitamins and minerals similar to the non-fermented ones, but they may also contain bioactive compounds and higher/more bioavailable amounts of certain minerals and vitamins), the potential probiotic properties of their constituent microorganisms affecting the gut and its interactions with bone/muscle/brain though production of derived metabolites, hormonal and immune modifications [14, 21] (Fig. 1). Nevertheless, it remains debatable if fermented dairy foods are indeed accompanied by health benefits that go beyond those of their non-fermented counterparts and if these mostly speculative in nature mechanisms are valid.

As such, the aim of the present work is to review and critically appraise the evidence from prospective and interventional studies on the consumption of fermented dairy products and specific outcomes related to musculoskeletal (bone health, muscle function, frailty, sarcopenia) and mental health (including cognition) in older adults and provide directions for future research. A special focus was placed upon identifying interventional studies that compared fermented vs. non-fermented dairy products on the selected outcomes.

## Methods

For the purposes of this narrative review, we conducted a literature search using MEDLINE database up to September 2023. We searched for longitudinal and interventional studies conducted in adults with age ≥ 60 years old or a mean cohort/participants’ age ≥ 65-year-old at follow-up. Relevant studies were selected using a combination of keywords for musculoskeletal health outcomes (bone, bone mineral density, osteoporosis, fracture, bone turnover or bone remodelling, sarcopenia, frailty, physical function or performance, muscle or lean mass, muscle strength, muscle function, muscle weakness, falls, daily activity disability), cognitive decline (cognition, cognitive, cognitive decline, cognitive impairment), depression (depression, depressive, depressive symptoms, depression-like behaviour, stress), and fermented dairy, yogurt, cheese, fermented or cultured milk, kefir or buttermilk as explanatory variables. Our goal was to identify studies comparing the consumption of fermented vs. non-fermented dairy products in relation to the selected outcomes. Due to the very limited number of such studies, we expanded our inclusion criteria to encompass: (i) prospective studies assessing the associations between fermented dairy product consumption and outcomes related to musculoskeletal and mental health, while also examining if similar or different associations were observed with the consumption of non-fermented dairy products and (ii) interventional studies investigating the effects of a fermented dairy product compared to a control. We excluded (i) studies that used as an intervention/ exposure total dairy food and made no distinctions between fermented and non-fermented dairy products or different dairy subtypes, (ii) studies that examined the effects of dairy drinks with probiotics that are not commercially available and/or present in typical diets (iii) studies that included fortified fermented dairy products vs. a non-fortified fermented dairy product only or vs. habitual diet only. Additional studies were identified by an extensive manual search of bibliography list in original papers and reviews. The relevance of studies was assessed by using a hierarchical approach based on title, abstract, and full manuscript. Abstracts and non-English papers were not included.

## Bone Health

### Observational Studies

Longitudinal studies that have assessed the associations between fermented dairy products and incidence of fractures in older adults, the most clinically important outcome in osteoporosis research, have resulted in mixed results [22–27] (Table 1). Hip fractures are the most debilitating osteoporotic fractures, they often require hospitalization and result in long-term disability in almost 1 in 2 patients and mortality in ~20% of the affected patients within a year of the hip fracture [28, 29]. In an analysis of two Swedish cohorts in middle-aged women and men with a mean follow-up of 20 years, higher intakes of fermented milk products (yogurt and soured milk) or cheese were associated with lower risks of all fractures and hip fractures in women only [23]. While these distinct results in women and men could be due to many factors including a greater susceptibility to fractures in women and sex differences in dairy consumption, it is worth mentioning that analyses in the Cohort of Swedish Men was based on a single exposure assessment, while in the Swedish Mammography Cohort on dietary data available from repeat questionnaires, potentially yielding stronger outcome associations in the women’s cohort. A similar favourable association between an increasing cheese consumption (average consumption based on multiple food frequency questionnaires (FFQs) administered at baseline and over the follow-up) and a lower hip fracture risk was shown by Feskanich et al. by using data form the Nurses’ Health Study in postmenopausal women who were followed for up to 32 years, however, this association did not persist after adjustments for main confounders [25]. No associations were found between yogurt consumption and hip fractures in women or between fermented dairy products (yogurt or cheese) and hip fracture rates in men [25]. In the Framingham Original Cohort, Sahni et al. found that medium to higher milk and yogurt consumers (> 1 serving/week) tended to have a lower risk of hip fractures compared to low consumers (≤ 1 serving/week) [24]. These associations appeared to be threshold dependent and driven by milk consumption, as there were not seen for yogurt consumption alone which was very low (0.4 servings/week). Likewise, no significant associations were observed between cheese and hip fracture risk. In a French cohort of community-dwelling older adults, individuals with low yogurt consumption (< 6 servings/week in men and < 7 in women) at baseline were at increased risk of fracture at the wrist, but not at the hip or the vertebrae [22]. Notably, the yogurt consumption of this French population was considerably higher than the respective consumption in other cohorts, which may explain the beneficial associations observed. No association was observed between low (vs. high) consumption of cheese or milk and fractures. Given that falls are the main cause of fractures, especially among older adults with osteoporosis, it is important to consider falls and fractures in parallel. Dietary factors can have skeletal, but also extra-skeletal effects (i.e., on muscles or neurological factors) that may alter fall risk and subsequent fracture risk [30]. Towards this end, a recent investigation using data from 2 cohorts of community-based older men and women, namely the Seniors-ENRICA cohort and the UK Biobank, found no associations between the consumption of yogurt or cheese at baseline and the risk of falls or fractures resulting from falls in either cohort, despite the fact that participants in the Seniors-ENRICA cohort had a higher consumption of total and fermented dairy than participants in the UK Biobank [27].

**Table 1:** Prospective studies on the associations of fermented dairy products and outcomes of musculoskeletal and mental health

Reference	Participants characteristics	Study & follow-up	Dietary assessment	Consumption of total dairy and fermented dairy products	Fermented dairy products (main results)	Non-fermented dairy products (main results)
Skeletal Health						
Feart et al., 2013 [22]	N = 1,482 (63% women), mean age: 76 y (68–95y)	Three-city (3C) study, France; FU: 8 y	FFQ (baseline)	Total dairy: men: 18.0 ± 7.8 serv/wk, women: 18.6 ± 7.8 serv/wk Yogurt: men: 5.8 ± 4.9 serv/wk, women: 8.0 ± 5.2 serv/week Cheese: men: 8.4 ± 4.7 serv/wk, women: 7.2 ± 4.5 serv/wk	**+/N** Low yogurt intake was associated with an ↑ risk of fracture at the wrist, but not at the hip or vertebrae. No association of low (vs. high) cheese consumption with fracture risk at any site	**N** No association of low milk consumption with fracture risk at any site
Michaëlsson et al., 2014 [23]	Swedish Mammography Cohort: N = 61,433 women (age: 39–74 y at baseline) Cohort of Swedish Men: 45,339 men (age: 45–79 y at baseline)	Swedish Mammography Cohort; Sweden; FU (median): 22 y Cohort of Swedish Men; Sweden; Sweden; FU (median): 13 y	Swedish Mammography Cohort: FFQ (baseline, ~ 10y FU) Cohort of Swedish Men: FFQ (baseline)	Total dairy: NR Yogurt: NR Cheese: NR	**+/N** Women with a ↑ intake of cheese or fermented milk products (yogurt and soured milk) had ↓ rates of all fractures and hip fracture compared with women with low intakes. There were no associations of cheese or fermented milk consumption with fracture rates in men	**N/−** A ↑ milk consumption was associated with ↑ risk of any fracture and hip fracture in women. No association between milk consumption and incident of all fractures or hip fractures in men
Sahni et al., 2014 [24]	N = 764 men and women, mean age: 77 y (68–96 y)	Framingham Original Cohort USA; FU: 11.6 y	FFQ (baseline)	Total dairy: NR Yogurt: 0.4 ± 1.3 serv/wk Cheese: 2.6 ± 3.1 serv/wk	**N** No association of yogurt or cheese consumption with hip fracture risk	**+** A trend towards a ↓ hip fracture risk with a medium/high milk intake *vs.* a low intake
Sahni et al., 2017 [31]	N = 628 (62% women), mean age: 75 y (65–96 y)	Framingham Original Cohort USA; FU: 3.9 y	FFQ (baseline)	Total dairy: vit D supplement users: 9.7 ± 8.0 serv/wk, non-users: 8.6 ± 7.0 serv/wk Yogurt: vit D supplement users: 0.6 ± 1.4 serv/wk, non-users: 0.3 ± 1.2 serv/wk Cheese: vit D supplement users: 2.7 ± 2.9 serv/wk, non-users: 2.4 ± 3.0 serv/wk	**+/N** No associations between yogurt or cheese and % change in BMD at FN, trochanter, or LS in the total population. or after stratification for use of vitamin D supplements (yes/no)	**+/N** No associations between milk or cream consumptions and % change in BMD in the total population. A ↑ milk intake was associated with ↓ bone loss at the trochanter among vitamin D supplement users only
Feskanich et al., 2017 [25]	Nurses’ Health Study (NHS): N = 80,600 women, mean age: 54 y at baseline Health Professionals Follow-up Study (HPFS) N = 43,306 men, mean age: 58 y at baseline	NHS; USA; FU: 20.8 y HPFS; USA; 17.5 y	FFQ (baseline and every ~ 4 years)	Total dairy: NR Yogurt: NR Cheese: NR	**+/N** An inverse association was found for cheese and risk of hip fracture in women only, which was attenuated after adjustments. Yogurt consumption was not associated with fracture risk in women or men	**+/N** Each serving of milk per day (cumulative average consumption) was associated with a significant 8% ↓ risk of hip fracture in a pooled analysis of men and women. Cream and ice cream were not associated with hip fracture risk
Michaëlsson et al., 2017 [26]	N = 61,240 women, age: 39–74 y at baseline	Swedish Mammography Cohort; Sweden; FU: 22 y	FFQ (baseline and after ~ 10 years)	Total dairy: NR Yogurt: NR Cheese: NR	**+** A ↑ fermented milk consumption was associated with ↓ rates of hip fracture	**−** A ↑ milk consumption was associated with ↑ rates of hip fracture
Biver et al., 2018 [32]	N = 482 women, mean age: 65 y	Geneva Retirees Cohort Switzerland; FU: 3.0	FFQ (baseline and follow-up)	Total dairy: NR Yogurt: NR Cheese: NR	**+/N** FDP consumption was associated with ↓ decreases of total and cortical vBMD, cortical thickness and area and ↓ increases in trabecular area (distal radius), but there were no significant associations for changes in trabecular vBMD and microstructure. No correlations at the tibia, or for changes in aBMD at the spine, hip, or radius. No associations between changes in vBMD or bone microstructure and ripened cheese consumption	**+/N** A ↑ milk consumption was associated with ↓ decreases in aBMD and failure load (radius)
Machado-Fragua et al., 2020 [27]	Seniors ENRICA (N = 2,981), mean age: 69 y UK Biobank (N = 8,927), mean age: 64 y	Seniors ENRICA, Spain; FU: 7.2 y UK Biobank, UK; FU: 10.2 y (median: 3.2 y)	Seniors ENRICA: FFQ (baseline) UK Biobank: ≥ 3 24-h recalls (baseline)	Total dairy: Seniors ENRICA: 2.3 (1.4) serv/day; UK Biobank: 0.9 (0.6) serv/day Yogurt: Seniors ENRICA: 0.6 (0.7) serv/day; UK Biobank: 0.4 (0.5) serv/day Cheese: Seniors ENRICA: 0.7 (0.9) serv/day; UK Biobank: 0.3 (0.3) serv/day	**N** No association of yogurt or cheese consumption with the risks of falls or risk of falls with fractures	**N/−** No association of milk consumption with the risks of falls or risk of falls with fractures in the Seniors ENRICA. Participants who consumed milk had a ↑ risk of falls vs. non-consumers in the UK Biobank
Muscle function/frailty/functional decline						
Vercambre et al., 2009 [33]	N = 4,809 women, age range: 76–82 y	E3N cohort, France; FU: 13 y	diet history questionnaire (baseline)	Milk and yogurt: 235 ± 192 g/day Cheese: 49 ± 39 g/day	**N** No association of cheese or milk + yogurt consumptions with functional impairment^1^	
Lana et al., 2015 [34]	N = 1,871 community-based men and women free of frailty, mean age: 69 y	Seniors ENRICA, Spain; FU: 3.5 y	diet history (baseline)	Total dairy: 306 ± 178 g/day Yogurt: 76 ± 83 g/day Cheese: 27 ± 35 g/day	**N** Non-significant associations of cheese, whole-fat or low-fat yogurt and incidence of frailty in fully adjusted models^2^	**+** A ↑ consumption of low-fat milk was associated with a ↓ risk of frailty (adjusted models)
Yoshida et al., 2019 [35]	N = 859, 40% women community-dwelling men and women without functional disability, mean age: 73 y	Hisayama Study, Japan; FU: 7 y	FFQ (baseline)	Total dairy: 111 (39–114) g/day Yogurt: NR Cheese: NR	**+/N** Yogurt consumption (vs. non-yogurt consumption) was associated with ↓ risk of functional capacity impairment^3^. Cheese consumers were at ↓ risk of developing functional capacity impairment^3^ vs. non-cheese consumers. Yogurt or cheese consumptions were not associated with ADL disability^4^	**N** The consumptions of milk, ice cream or milk-based drinks were not associated with functional capacity impairment^3^ or ADL disability
Rahi et al., 2021 [36]	N = 823, non-institutionalized, frail-free older adults (65% women), mean age: 73 y	3C-study, France; FU: 10 y	FFQ (baseline)	Total dairy: 2–4 times/d: 53% of the population Yogurt: 0.5–1.5 times/d: 51% of the population Cheese: 0.5–1.5 times/d: 54% of the population	**+/N** Non-significant associations of fresh dairy (yogurt and cottage cheese) or cheese consumptions and incidence of frailty (definition 1).^5^ High vs. low consumers of fresh dairy products had a lower incidence of frailty (definition 2)^6^	**N** No significant associations of milk consumption and incidence of frailty (using two different definitions)^5,6^
Siefkas et al., 2022 [37]	N = 2,524 non-frail adults (55% women), mean age: 60 y	Framingham Offspring Study, USA; FU: 11.3 y	FFQ (baseline)	Total dairy: 9.6 ± 6.9 serv/wk Yogurt: 0.2 [0.0–1.5] serv/wk Cheese: 2.8 ± 3.0 serv/wk	**+/N** A ↑ yogurt intake was associated with ↓ odds of frailty^2^ and ↑ follow-up gait. Cheese consumption was not associated with frailty	**N** Milk consumption was not associated with frailty
Mental health						
Vercambre et al., 2009 [33]	N = 4,809 women, age range: 76–82 y	E3N cohort, France; FU: 13 y	FFQ (baseline)	Milk and yogurt: 235 ± 192 g/day Cheese: 49 ± 39 g/day	**N** No association of cheese or milk + yogurt consumptions with recent cognitive decline^7^	
Dobreva, et al. 2022 [38]	N = 249,511 (53% women), mean age: 62 y	UK Biobank, UK; FU: 10.2 y (median: 3.2 y)	FFQ (baseline)	Total dairy: NR Yogurt: NR Cheese: 2–4 times/week: 45% of the population	**+** Consumption of cheese once/week was associated with a ↓ dementia risk vs. no consumption. This association did not remain significant in a fully adjusted model for all covariates and food groups	
Hockey et al., 2022 [39]	N = 2,603 men, mean age: 54 y	Kuopio Ischaemic Heart Disease Risk Factor Study, Finland; FU: 24 y	4-day food records (baseline)	Total dairy intake: 683 (452, 927) g/d Fermented dairy intake: 103 (24, 282) g/d Nonfermented dairy intake: 473 (264, 727) g/d	**+** Those in the highest (vs. the lowest) tertile of FDPs (excluding cheese) had a ↓ depression risk	**−** Those in the highest (compared with lowest) tertile of non-FDPs had a ↑ risk of depression diagnosis
Ni et al., 2022 [40]	N = 4,668 older adults at high CVD risk (48% women), mean age: 65 y	PREDIMED-Plus study, Spain, FU: 2 y	FFQ (baseline)	Total dairy: 284 (207, 413) g/day Yogurt: NR Cheese: NR	**N** In fully adjusted models, consumption of yogurt, cheese or total FDPs were not associated with changes in cognitive function	**−** A ↑ total and whole-fat milk consumption were associated with ↑ global cognitive decline

Main results are presented with ( +) if beneficial associations, (N) if neutral associations and (−) if unfavourable associations

*aBMD* areal bone mineral density, *CVD* cardiovascular disease, *FFQ* food, frequency questionnaire, *FDP* fermented dairy products, *FN* femoral neck, *FU* Follow-up, *LS* lumbar spine, *vBMD* volumetric BMD, *NR* Not reported, *serv/wk* servings/week, *y* year, ↑ greater, ↓ lower

^1^Functional impairment defined as a score > 0 on simplified IADL scale. ^2^frailty defined according to the criteria by Fried et al. ^3^functional capacity impairment defined as a score of ≤ 12 on Tokyo Metropolitan Institute of Gerontology Index of Competence, which consists of 13 questions about instrumental activities of daily living (IADL), intellectual activity, and social roles, ^4^ADL disability defined as a Barthel Index score of ≤ 95, ^5^frailty defined according to the Cardiovascular Health Study frailty index, ^6^FRAIL scale, ^7^recent cognitive decline defined as a score < 33 based on the ‘DEtérioration Cognitive Observée’ (observed cognitive deterioration) (DECO) scale

Taken together, existing studies support favourable or neutral association between fermented dairy products and incident fractures, with the discrepancies in the results being potentially related to differences in study design (e.g., follow-up duration, single or repeated dietary assessments over the follow-up), population characteristics (e.g., age, sex, BMI status), consumption levels of fermented dairy products (e.g., these seem to be higher in European countries than in the US) and incident rates of fractures.

Two studies explored the association of fermented dairy products with age-associated bone loss [31, 32]. In an analysis of the Framingham Original Cohort, no significant associations were observed between fermented (yogurt or cheese) or non-fermented (milk or cream) types of dairy foods and percentage of change in BMD over the 4-year follow-up at any site (femoral neck, trochanter, and lumbar spine) in the total population. Further analysis according to the use of vitamin D supplements revealed that a higher consumption of dairy (milk, yogurt and cheese) was significantly associated with attenuated reductions of trochanteric BMD among individuals using vitamin D supplements only [31]. Specifically, greater consumptions of cheese and milk tended to be protective against bone loss at clinically relevant sites in vitamin D supplement users only, whilst no associations were observed for yogurt consumption. Collectively, these findings do not support distinct associations for fermented and non-fermented dairy foods but suggest beneficial associations with a higher combined consumption of fermented and non-fermented dairy foods among individuals who use vitamin D supplements. Those not taking vitamin D supplements may miss these benefits. These findings could possibly be explained by the biological role of vitamin D in calcium absorption and skeletal homeostasis.

Further extending these results, Biver et al., investigated the associations of fermented and non-fermented dairy products with age-related changes in areal BMD (aBMD) at clinically relevant skeletal sites, but also changes in bone microstructure and strength at the peripheral skeleton (*i.e.,* distal radius and tibia) [32]. The consumption of fermented dairy products (yogurt, fresh cheese, and fermented milk) was associated with attenuated cortical bone loss at the non-weight bearing radius at the 3-year follow-up. In line with the findings of the Framingham Original Cohort, these associations were more pronounced among users of vitamin D supplements. There were no significant associations of consuming fermented dairy products with changes in bone microstructure or strength at the weight-bearing tibia, or for changes in areal BMD (aBMD by DXA) at the spine, hip, or radius. Consuming ripened cheese was not associated with skeletal benefits at any site. Notably, a greater milk consumption was also associated with less pronounced decreases in aBMD and failure load at the distal radius. These findings further support that the protective associations observed were not unique to fermented dairy products.

### Clinical Trials

Few RCTs with a short duration (≤ 2 months) have assessed the consumption of fermented dairy products on bone-related hormones and bone turnover markers (BTMs) reflecting bone formation or bone resorption as surrogate markers of bone health [41] (Tables 2, 3). Assessment of BTMs prior and after few weeks or months of an intervention can capture early changes in bone metabolic activity before established changes in bone structure. Their assessment is clinically relevant as BTMs have been shown to predict the rate of bone loss and to a lesser extent the longer-term risk for fragility fractures. In a cross-over randomized controlled trial, Heaney et al. compared the effects of yogurt and jelled fruit-flavoured snack on N-terminal telopeptide of type 1 collagen (NTx-a marker of bone resorption) concetrations, in postmenopausal women with habitually low calcium intake [42]. The authors reported a significant reduction in NTx levels following 7–11 days of intervention of 3 servings of yogurt daily (providing 570 kcal, 21 g of protein and 900 mg of calcium) compared with the consumption of the jelled snack. Notably, the two interventions were not matched for their caloric content, the amount of yogurt per serving was not specified and the duration of the intervention was short and variable between participants. In another study, older individuals with suboptimal 25-hydroxy vitamin D levels (< 30 ng/mL) were randomized to receive vitamin D-fortified cheese (600 IU/d), non-fortified cheese, or no cheese (control) for 2 months [43]. No specific details on the type of cheese were provided. There were no within-group changes or differences in parathyroid hormone (PTH), or osteocalcin responses between those who consumed the non-fortified cheese and those in the control group.

**Table 2:** Interventional studies on the effects of fermented dairy products and outcomes of musculoskeletal and psychocognitive health

Reference	Design	Population	Duration	Intervention	Control	Outcomes	Main finding
Skeletal health							
Heaney et al., 2002 [42]	randomized, crossover design	N = 29 postmenopausal women not taking Ca supplements with dietary Ca intake (< 600 mg/d)	7–11 days	fruit- flavoured yogurt (3 servings/day providing 570 kcal, 21 g of protein and 900 mg of calcium)	jelled fruit-flavoured snack	NTx, urine Ca	NTx was ↓ after yogurt consumption
Johnson et al., 2005 [43]	partially double-blind RCT	N = 100 older individuals, age ≥ 60 y	2 months	-non-fortified cheese (85 g/day) -vit D fortified (600 IU/d) cheese (85 g/day)	no cheese	BTMs and bone-related hormones: 25-OHD, PTH and OC	No differences in PTH, or OC between groups. The vit D-fortified cheese group had ↑ baseline 25-OHD levels and experienced a greater ↓in 25-OHD than other groups
Muscle and cognitive function							
Alemàn-Mateo et al., 2012 [44]	randomized, parallel groups	N = 40 sarcopenic older men and women, mean age 76 y	3 months	ricotta cheese (210 g/day) in addition to habitual diet	habitual diet	aSMM by DXA, hand grip strength	No group differences in the % relative change in aSMM or lean body mass. The intervention group tended to maintain their hand grip strength compared to ↓seen in controls (p = 0.06)
Alemàn-Mateo et al., 2014 [45]	randomized, single blind, parallel groups	N = 100 healthy, non-sarcopenic older men and women, mean age: 70 y	3 months	ricotta cheese (210 g/day) in addition to habitual diet	habitual diet	aSMM by DXA, hand grip strength, SPPB (balance score, gait speed, five chair rise), SCPT	aSMM and aSMMI ↑ in the intervention group, but ↓ in the control group. The relative %change in strength ↓in both groups, with a trend towards lower ↓ in the intervention group (p = 0.07). No differences in the % relative change in SPPB or SCPT between groups, although balance score (in SPPB) ↑ in the intervention group but ↓ in the control group (group differences, p = 0.05)
Bagheri et al., 2021 [46]	randomized, double blind, placebo-controlled parallel groups	N = 30 healthy untrained older men, mean age: 68 y	8 weeks	Icelandic yogurt (200 g/day) and resistance exercise (3x/week)	iso-energetic placebo (carbohydrate-based pudding) and resistance exercise (3x/week)	lean mass by BIA, upper body strength by bench press, lower body strength by leg press)	The intervention group experienced greater ↑ in lean mass, bench press and leg press compared to the control group
Suzuki et al., 2019 [47]	RCT, crossover design	N = 71 community-dwelling older women with mild cognitive impairment, age ≥ 70 y	3 months	mold-fermented (MFC) camembert cheese (2 × 16.7 g/day)	non-MFC (processed cheese made from mozzarella and cream cheese) (2 × 16.7 g/day)	grip strength, knee extension strength, and usual walking speed, GDS, MMSE	No significant group x time interactions for grip strength, knee extension strength, usual walking speed, maximum walking speed, GDS or MMSE

*aSMM* appendicular skeletal muscle mass, *ASMMI* ASMM index, *BAP* bone alkaline phosphatase, *BIA* bioelectrical impedance, *BTMs* bone turnover markers, *CTX* C-terminal telopeptide of type 1 collagen, *DXA* dual-energy X-ray absorptiometry, *GDS* Geriatric Depression Scale, *MFC* mold-fermented cheese, *MMSE* Mini-Mental State Examination, *NTX* N-terminal telopeptide of type 1 collagen, *OC* osteocalcin, *PTH* Parathyroid hormone, *RCT* randomized controlled trial, *SPPB* short physical performance battery, *SCPT* stair-climb power test, *y* year, *25OHD* 25-hydroxy-cholecalciferol, ↑ increase, ↓ decrease

**Table 3:** Summary of interventional studies on the effects of fermented dairy products and outcomes of musculoskeletal and mental health

	Skeletal health		Muscle health		Mental health	
	N studies	Results	N studies	Results	N studies	Results
FDP + habitual diet vs habitual diet	N = 1	No effects on bone turnover markers	N = 2	No or beneficial effects on appendicular skeletal muscle mass, muscle strength and some physical performance tests	N = 0	–
FDP vs non-FDP	N = 0	–	N = 0	–	N = 0	–
FDP vs. non-dairy	N = 1	Some beneficial effects (reduction in bone resorption)	N = 1	Some beneficial effects on lean mass and muscle strength	N = 0	–
Comparison of different FDPs	N = 0	–	N = 1	No differences	N = 1	No differences

*FDPs* fermented dairy products

Collectively, very limited evidence suggests that fermented dairy products may offer short-term protection against bone breakdown in older individuals, but it remains uncertain whether these bone metabolic changes translate to beneficial effects on BMD or reduction of fracture.

## Muscle/Functional Decline and Frailty

### Observational Studies

We considered longitudinal studies that assessed the associations between fermented dairy products and the risks of sarcopenia, frailty and the accompanying functional impairment or limitations/disability in performing activities of daily living (ADL) or changes in associated parameters (e.g., muscle strength) [33–37, 48] (Table 1). Nevertheless, our literature search did not retrieve any prospective studies on sarcopenia. In a sample of Spanish older adults enrolled in the Seniors-ENRICA cohort, Lana et al., examined the associations between consumption of dairy products and subtypes (at baseline) and risk of frailty using the Fried criteria (exhaustion, weakness, low physical activity, slow walking speed, unintentional weight loss) [34]. No significant associations were observed for the intakes of cheese, whole-fat or low-fat yogurt after adjustments for main confounders. A higher consumption of low-fat milk and a higher combined consumption of low-fat milk and yogurt were associated with lower risk of frailty and, specifically, of slow walking speed and weight loss. Rahi et al. investigated the prospective associations between fresh dairy products (yogurt and cottage cheese) and cheese with incidence of physical frailty (Cardiovascular Health Study frailty index) in non-frail individuals [36]. When comparing the participants with the lowest consumption with those with the greatest consumption, neither fresh dairy nor cheese consumption was associated with the risk of physical frailty in fully adjusted models. In a sensitivity analysis using another definition of frailty (as assessed by the FRAIL scale) that includes fatigue and weight loss, but also assesses participants’ underlying diseases and perceived ability to walk and climb stairs, those with high consumption of fresh dairy products were at lower risk of developing frailty than those with low consumption, whilst no associations were found for high (vs. low) consumers of milk and cheese. Even though this second definition identified fewer frail individuals and the 3-way comparison (low, intermediate, high consumers) did not reach significance, these results point towards a protective association between fresh dairy products and frailty, which was not observed with other fermented (cheese) or non-fermented (milk) dairy products. Finally, in the Framingham Heart Study, a higher yogurt intake was modestly associated with reduced frailty onset (Fried criteria) and individual frailty components (i.e., gait speed and physical activity) despite the low yogurt consumption at population level (0.2 servings/week). No associations were observed for cheese or milk [37]. It is important to mention that although there is consensus on the importance of detecting sarcopenia and frailty as early as possible, their definition and diagnostic criteria vary considerably in the literature, making challenging to appreciate the impact of dietary factors as preventive/treatment strategies for these conditions.

Few further studies have explored the consumption of fermented dairy products in relation to functional impairment or disability in performing activities of daily living (ADL). Impaired functional capacity is established as an early sign of functional disability and progresses to ADL disability as underlying disease/conditions exacerbate. In a prospective cohort study of community-based Japanese older individuals without functional disability at baseline, Yoshida et al. examined the contribution of yogurt and cheese consumption at baseline to the decline of functional capacity (as assessed by the Tokyo Metropolitan Institute of Gerontology Index of Competence) and ADL (as assessed by the Barthel Index) [35]. Those consuming yogurt had a 14% lower risk of functional capacity impairment compared to non-yogurt consumers. Similarly, cheese consumers were at lower risk of developing functional capacity impairment than non-cheese consumers. Neither yogurt nor cheese consumptions were associated with the development of ADL disability. These findings may reflect the relatively short follow-up period which might have been insufficient to evaluate such an association or the increasing contributions of other factors (e.g., disease) to ADL disability. The consumptions of non-fermented dairies (milk, ice cream or milk-based drinks) were not associated with functional capacity impairment or ADL disability. In line with these results, the findings of an earlier longitudinal study with a 13-year follow-up support no associations between the combined milk + yogurt intake or cheese intake and the risk of functional decline in performing instrumental ADL [33]. Null associations between fermented (yogurt or cheese) or non-fermented (milk) dairy intake and incident risk of functional disability were also reported in a recent prospective cohort study in older Japanese individuals followed up for ~ 8 years [48].

### Clinical Trials

Two RCTs used the same intervention protocol to explore the effects of ricotta cheese on muscle mass, strength, and physical performance in community-dwelling sarcopenic [44] and non-sarcopenic [45] older adults. Ricotta is a soft, fermented cheese that is traditionally made from the leftover whey from cheesemaking and literally means “recooked/refined”. In a first RCT, sarcopenic older women and men were instructed to consume 210 g/day of ricotta cheese in addition to their habitual diet or to follow their habitual diet (control) for 3 months [44]. The intervention group tended to maintain their grip strength compared to reductions seen in controls, with no further differences in relative changes in appendicular skeletal muscle mass between groups.

In a second RCT by the same research group and by using the same protocol, older adults free of sarcopenia and physical disability were allocated to the intervention group (210 g ricotta cheese + habitual diet) or the control group (habitual diet) [45]. After 12 weeks of intervention, appendicular skeletal muscle mass was maintained/increased in the intervention group, whilst it decreased in the control group. Grip strength relative to body weight was reduced in both groups although these reductions tended to be greater in the control group. Relative changes in balance test scores were positive in the ricotta cheese + habitual diet group and negative in controls. No significant changes were observed in short physical performance battery (SPPB) and stair-climb power test. Notably, this intervention involved the consumption of a substantial amount of cheese which may significantly alter the overall dietary habits of the older participants; future studies are needed to confirm adherence to such a dietary change in other cultural settings.

In a recent study, healthy untrained older males were randomized to receive Icelandic yogurt or an iso-energetic placebo immediately after performing a resistance training program (3 × /week) for 8 weeks [46]. Participants in the Icelandic yogurt group experienced greater increases in body mass, lean mass and muscle strength assessed by bench press and leg press compared to those receiving placebo, whilst no changes in fat mass were observed in either group. These results suggest that a generally feasible dietary intervention can enhance exercise benefits towards the prevention of sarcopenia and falls and promotion of independent living during ageing. However, replication studies are needed in other populations of older adults.

Finally, Suzuki et al., investigated the effects of mold-fermented cheese (MFC: camembert cheese) vs. a non-MFC (processed cheese made from mozzarella cheese and cream cheese) on physical function in community-dwelling older Japanese women [47]. The rationale for such a comparison was that the camembert cheese, and specific ingredients produced by this type of ripening, have anti-inflammatory effects and thus, may protect against ageing-related declines in physical function. This hypothesis was not confirmed in that study, as after 3 months of intervention there were no differences between groups in grip strength, knee extension strength, and usual walking speed.

### Cognitive Decline and Psychological Health

A large body of research suggests, and confirms in some cases, the direct role of dietary components (nutrients or foods) and patterns on cognitive status in older adults [49]. However, evidence on the associations between fermented dairy products consumption and cognitive performance is limited and provides conflicting results. In specific, although the results of cross-sectional studies are supportive of a positive (beneficial) association between fermented dairy products intake, especially of cheese intake, and total or specific cognitive functions [50–52], longitudinal studies do not reveal such an association (Table 1).

Data from the UK Biobank cohort initially showed that consumption of cheese once a week decreased dementia risk compared to no consumption, even after adjustment for sociodemographic, lifestyle, physical and mental health factors [38]. However, when all food groups and further covariates were included in the model, the association became non-significant. Similarly, in the PREDIMED-Plus trial in Spanish older adults at high cardiovascular disease risk, the associations between fermented dairy products with global cognitive function were not significant after adjustment for confounders. [40]. Hence, both reports indicate that important intercorrelations between foods patterns or diet and lifestyle patterns may be involved in the relationship between consumption of fermented dairy foods and cognitive changes.

There is also one RCT in this area that investigated the potential effect of the twice a day consumption of the MFC camembert cheese, on the brain-derived neurotrophic factor, compared to the consumption of a processed cheese made from mozzarella and cream cheese [47] (Tables 2, 3). The brain-derived neurotrophic factor has been previously shown to exert protective effects on neuronal circuitry involved in Alzheimer’s disease [53]. The analyses of the RCT indicated that the levels of this factor significantly increased after the consumption of the MFC camembert cheese but not after the consumption of the control cheese. However, no significant changes were observed in the 3 categories of the Mini-Mental State Examination (MMSE) score (normal, mild cognitive impairment, moderate or severe cognitive impairment), or in the depression score, indicating that the improvements observed in the biological marker were not translated into clinical outcomes, at least, after this short-term, 3-month, intervention.

Regarding psychological health, the evidence on the potential role of fermented dairy intake in depression is even scarcer. There is only one report from a population-based study concluding that higher fermented dairy intake (assessed by 4-day food records) at middle age was associated with lower odds of having elevated depressive symptoms (assessed by the 18-item Human Population Laboratory depression scale) and a lower risk of depression diagnosis [39]. Notably, the mean follow-up was 24 years and dietary intake was assessed only at baseline. Interestingly, the association was found only after excluding cheese intake from the fermented dairy products group. The authors hypothesized that cheese has higher sodium content compared to yogurt, kefir, buttermilk, sour milk; this sodium content is linked to a higher risk of cardiovascular disease, which may in turn increases predisposition to develop depression. Another explanation could be the potential connection between cheese consumption and other lifestyle patterns with an impact on depressive symptomatology.

## Discussion

Fermented dairy products are low-cost, widely available and, generally, well-accepted foods which can make important contributions to reaching recommendations for several nutrients (protein, calcium, magnesium) [19, 20]. They do not require cooking or need only minimal preparation and most of them, have a soft texture, with these characteristics making them a practical option for older adults (even for those with chewing and/or swallowing difficulties). Fermented dairy products may be better tolerated than the non-fermented ones in older individuals with lactose intolerance, and they may further provide viable microorganisms with potential important biological roles. In this review, we aimed to investigate whether fermented dairy products offer benefits against musculoskeletal and psychocognitive ageing and how such benefits compare to the effects/associations observed with consumption of non-fermented dairy foods.

Prospective studies support favourable or neutral associations of fermented dairy products with musculoskeletal health, and rather neutral associations with psychocognitive outcomes in older individuals. Given that in some studies there were associations of same direction/magnitude for milk, current evidence does not support that the potential small benefits observed with fermented dairy products go beyond those of non-fermented dairy products. These mixed results for fermented dairy products may reflect small/null effects in this population group, but they may also be due to other factors and methodological limitations. Different population may have different fermented and non-fermented dairy products consumption levels, or they consume them in different contexts. For example, mean consumptions of yogurt and cheese and their relative contributions to total dairy intakes differed greatly in cohorts (as an example in France vs. the USA [22, 24]). Given there may be threshold effects on different outcomes, absence of associations may be explained by low consumptions. Other cultural considerations include (i) the degree of processing of dairy products which may impact fermentation-related constituents, (ii) their consumption in raw/cooked form (e.g., in a pizza) and the potential destruction of live microbes in the latter case (without excluding though the possibility that these inanimate microorganisms and/or their components may still confer a health benefit [4, 6]), (iii) fortification of dairy foods (mostly milk) with vitamin D in some countries [37] and (iv) the habitual consumption of fermented dairy products with other foods (whose nutrients may exert synergistic or antagonistic health effects), making it difficult to disentangle the net effects of fermented dairy products from those of added nutrients, non-fermented dairy products, or dietary patterns.

Most of the observational studies assessed the consumption of fermented dairy products using FFQs, which, may be less accurate than other dietary assessment methods (i.e., food records) in measuring the consumption of specific dairy products with low intake at a population level but high intake in some individuals. Another source of bias in nutrition research is the over- and underreporting of dietary intakes, particularly among older individuals, which could lead to misclassification of fermented dairy food consumption. To reduce this measurement error, some of the included studies used the energy-adjusted residual method [24, 27, 31, 37, 40]. The lack of repeated assessments of dietary intake and of fermented dairy products, in specific, over the follow-up is a further limitation of most prospective studies in our review (Table 1). Older participants may change their fermented dairy food consumptions because of lactose intolerance/maldigestion, disease or health awareness, or as a result of the introduction of other products to the markets (e.g., plant-based alternatives).

There was considerable variation in the definition and reporting of fermented dairy products serving size, with some studies using the term “servings/day”, “portions/day” or “grams/day”. Similarly, considerable variation was observed in the reporting of outcome variables related to bone (e.g., types of fractures considered, BMD at different skeletal sites), muscle/physical function (i.e., differences in frailty and sarcopenia assessments and definitions), cognition (i.e., different domains of cognition considered, using several instruments for their evaluation) making it difficult to compare research findings and possibly to detect some small effects. Future studies in this research area should include a set of well-defined exposure and outcome variables, assessed preferably by health care professionals to reduce error, and reported in a consistent format (see Box 1 for directions for future research).

**Box 1:** Directions for future research

Prospective studies should:
•Assess the consumption of fermented, but also non-fermented dairy products at multiple time points
•Consider a uniform approach to allocate and quantify (i.e., standardize serving sizes) fermented dairy foods
•Include long-term follow-ups to allow the detection of hard outcomes (e.g., incidence of fractures, falls, frailty/sarcopenia or dementia)
•Use thorough assessments of bone, muscle and psychocognitive health using validated tools and universal definitions
•Identify the timing/onset of exposure to fermented dairy foods (e.g., middle-adulthood, even late adulthood) that may be mostly/more consistently associated with favorable outcomes in older adults
•Address/consider important confounding factors including demographics, comorbidities and medication use and lifestyle factors including consumption of non-fermented dairy, other dietary factors, smoking and alcohol consumption and physical activity
RCTs should:
•Have well-designed methodologies, sufficient numbers of participants to detect meaningful changes/differences and longer durations
•Describe in detail the experimental products
•Provide insights into how added nutrients (e.g., added sugars) or the food matrix may influence the outcomes of interest
•Detail compliance and challenges related to their consumption
•Explore ways to optimize the benefits from their consumption (e.g., are benefits attenuated when fermented dairy products are consumed in cooked form? combinations with physical activity?)
•Assess whether fermented dairy products are more beneficial than non-fermented dairy products for musculoskeletal and psychocognitive health or whether the consumption of live bacteria as part of food matrix or in the form of a supplement (e.g., probiotics) exert differential effects on relevant outcomes
•Extend the investigation to the role of functionally fortified fermented dairy products (good vehicles to deliver nutrients and bioactive compounds)
•Differentiate the effects of fermented dairy products for prevention or treatment of musculoskeletal and cognitive disorders and identify relevant settings (e.g., community-based vs. institutionalized individuals)
•Elucidate mechanistic pathways

In addition to epidemiological data, very few available interventional studies suggest overall favourable effects of fermented dairy products (yogurt or cheese) on musculoskeletal health, while studies reporting psychcognitive outcomes are largely lacking in older adults (Tables 2, 3). With regards to bone, only one study suggested that fermented dairy products may offer acute protection against bone breakdown (assessed by BTMs) in older individuals, in particularly those with low baseline calcium intake [42], whilst it remains still uncertain whether bone metabolic changes translate to beneficial effects on BMD, bone microstructure or reduction of fracture risk. Incorporating fermented dairy products into habitual diet or combining them with exercise may also have some benefits for muscle mass and performance [44–46]. Notably, available studies are highly diverse in terms of duration (~ 10 days to 3 months), the type of fermented dairy products under investigation, the relative contribution of fermented to total dairy intake and outcome assessment, making it very difficult to compare results across studies conducted in different settings and populations (Table 2). Thus, future studies with longer duration and larger sample sizes are required to confirm the causal effect of fermented dairy products on musculoskeletal and cognitive outcomes in older adults and elucidate their effect in different subgroups including men and women, individuals with and without osteoporosis/ sarcopenia/ cognitive impairment, those with poor dietary intakes and those living in the community or in care homes (Box 1).

An important point of discussion is the choice of an appropriate control group. Some of the interventions used the habitual diet as a control intervention [43–45]. Although such a design allows to separate the effects of treatment, it is not informative on whether the observed effects are attributable to the increased consumption of fermented dairy products per se, or to associated changes in the consumption of other foods/food groups (i.e., displacement effects). Some other studies utilized a replacement food partially or fully matched with a fermented dairy food for their caloric content, thus providing evidence that yogurt can have beneficial effects on bone and muscle compared to non-dairy foods [42, 46]. Depending on the research question, relevant control groups may include: (i) a non-fermented dairy control (i.e., do fermented vs. non-fermented dairy products have differential effects on outcomes?), (ii) another fermented dairy product (e.g., is yogurt more beneficial for a specific health outcome than cheese, or do two cheeses produced from different fermentation/ripening processes have differential effects on outcomes?) or (iii) a component of a fermented dairy product (e.g., do live bacteria have differential effect on outcome depending on whether they are supplied as part of a food matrix such as yogurt or in the form of a probiotic supplement?) (Box 1).

The focus of the present review was to identify studies comparing the consumption of fermented versus non-fermented dairy products in relation to the selected age-related health outcomes. We identified a very limited number of such studies and thus, further included prospective studies assessing the associations between fermented dairy product consumption and outcomes related to musculoskeletal and mental health and ii) interventional studies investigating the effects of a fermented dairy product compared to a control. Hence, the results of our review should be interpreted with this limitation in mind. Further, we focused on studies that investigated the effects of fermented dairy products on outcomes of interest and did not consider interventions with total dairy products which made no distinctions between fermented and non-fermented dairy products. Notably, in a large RCT in older adults residing in care homes (N = 7195), an increase in total dairy products resulted in reductions in fractures by 33% (similar to the effects of antiresorptive drugs in people at high osteoporotic risk) and falls by 11% compared to habitual diet [54]. These findings suggest that the provision of dairy products, including the fermented ones, may be an appropriate and relatively simple intervention for the prevention/treatment of musculoskeletal diseases in older individuals and underpin the need for future RCTs to further explore whether dairy product subtypes (e.g., fermented vs. non-fermented dairy products) have differential effects on musculoskeletal parameters (Box 1). Similarly, we excluded studies that compared a fortified fermented dairy product vs. continuation of habitual diet as they do not allow to distinguish the effects of fortified nutrients (vitamin D, calcium) from their combined effects with the conventional/non-fortified fermented dairy products [55, 56]. Furthermore, we applied strict age inclusion criteria, thus, studies in slightly younger individuals or younger groups at risk were excluded.

## Conclusions

In conclusion, prospective studies support either favourable or neutral associations of fermented dairy products with outcomes of musculoskeletal health or neutral associations with mental health outcomes in older adults. Few interventional studies suggest overall favourable effects of fermented dairy products (yogurt or cheese) on musculoskeletal health, while studies reporting cognitive outcomes are largely lacking for this age group. Given the small number of studies identified and the even fewer studies evaluating the impact of fermented vs. non fermented dairy foods on the reported health outcomes, it remains challenging to formulate recommendations regarding amounts and types of fermented dairy products that may offer protection or determine whether there are added benefit to fermented dairy food consumption. The studies presented in this review provide preliminary evidence that can be used to extend the research base on fermented dairy products in relation to musculoskeletal and mental health ageing.
